# Acute cholecystitis with massive upper gastrointestinal bleed: A case report and review of the literature

**DOI:** 10.1186/1471-230X-7-12

**Published:** 2007-03-26

**Authors:** Sundeep S Saluja, Sukanta Ray, Manpreet S Gulati, Sujoy Pal, Peush Sahni, Tushar K Chattopadhyay

**Affiliations:** 1 Department of Gastrointestinal surgery, Room No 1005, PC block, 1^st ^floor, AIIMS, Ansari Nagar, New Delhi, 110029, India; 2 Department of Radiology Ground floor, AIIMS, Ansari Nagar, New Delhi, 110029, India

## Abstract

**Background:**

Cystic artery pseudoaneurysm is a rare complication following cholecystitis. Its presentation with upper gastrointestinal hemorrhage (UGIH) is even rarer. Thirteen patients with cystic artery pseudoaneurysm have been reported in the literature but only 2 of them presented with UGIH alone.

**Case presentation:**

We report a 43-year-old woman who developed a cystic artery pseudoaneurysm following an episode of acute cholecystitis. She presented with haematemesis and melaena associated with postural symptoms. Upper gastrointestinal endoscopy revealed a duodenal ulcer with adherent clots in the first part of the duodenum. Ultrasonography detected gallstones and a pseudoaneurysm at the porta hepatis. Selective hepatic angiography showed two small pseudoaneurysms in relation to the cystic artery, which were selectively embolized. However, the patient developed abdominal signs suggestive of gangrene of the gall bladder and underwent an emergency laparotomy. Cholecystectomy with common bile duct exploration along with repair of the duodenal rent, and pyloric exclusion and gastrojejunostomy was done.

**Conclusion:**

This case illustrates the occurrence of a rare complication (pseudoaneurysm) following cholecystitis with an unusual presentation (UGIH). Cholecystectomy, ligation of the pseudoaneurysm and repair of the intestinal communication is an effective modality of treatment.

## Background

Cholelithiasis has a high prevalence in Northern India. Only one-third of patients detected to have gallstones are symptomatic [1]. The usual presentation varies from biliary colic to gallstone associated pancreatitis. However, massive upper gastrointestinal haemorrhage (UGIH) following an episode of acute cholecystitis is rare and only a few case reports are available in the literature [2-4]. We report our experience of managing a patient with this rare complication of acute cholecystitis.

## Case presentation

A 43-years-old woman presented to the emergency services of the All India Institute of Medical Sciences, New Delhi with a history of haematemesis and melaena along with postural symptoms. She gave no history of abdominal pain, fever or jaundice. She had been diagnosed to have acute cholecystitis a week before presenting to us and had been managed conservatively with antibiotics (ciprofloxacin 500 mg twice a day for 7 days) and anti-inflammatory analgesics. She was a known hypertensive on medical treatment. On examination she had tachycardia of 110/min and blood pressure of 100/60 mmHg. General physical examination showed marked pallor but no icterus. She had tenderness in the right upper quadrant (RUQ) on deep palpation.

At admission her haemoglobin was 4.5 g/dl (10–15 g/dl), total leucocyte count 32.4 × 10^3 ^cells/cc (4–11 × 10^3 ^cells/cc), platelet count 3.78 × 10^5^cells/cc (1.5–4 × 10^5 ^cells/cc) and prothrombin time was 4 seconds prolonged (control: 14 seconds). Her liver function tests showed a bilirubin of 2.0 mg/dl (0.8–1.0 mg/dl), ALT 85 IU (0–50 IU), AST 40 IU (0–50 IU) and alkaline phosphatase of 497 IU (80–240 IU).

She was resuscitated with intravenous fluids, blood transfusions (4 units) and started on parenteral proton pump inhibitors. She then underwent an upper gastrointestinal endoscopy (UGIE), which showed that the oesophagus was normal, the stomach was full of blood and blood clots, a deep ulcer (1.5 cm) was seen on the posterior inferior surface of the first part of the duodenum with adherent clots. The second part of the duodenum was normal and contained bile.

**Figure 1:**
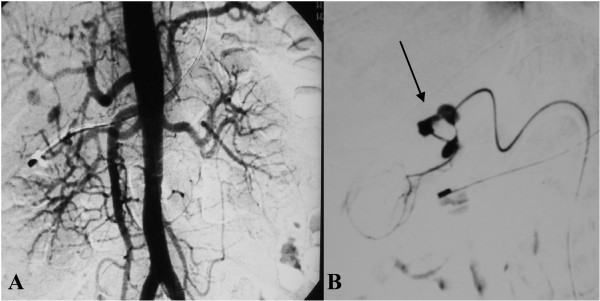
A: Flush aortogram showing pseudoaneurysms in the coeliac artery territory. B: Selective hepatic artery angiogram showing two small pseudoaneurysms (arrow) in relation to the cystic artery.

**Figure 2:**
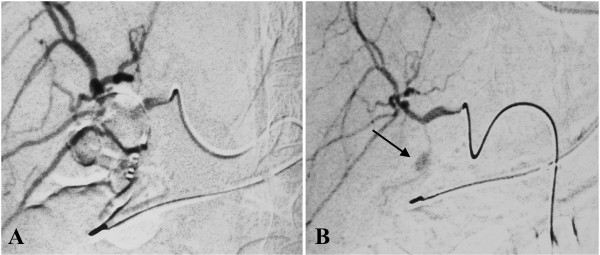
A: Angio-embolization of the pseudoaneurysm with gelfoam and microcoil. B: Post embolization image showing non-filling of the pseudoaneurysm (arrow).

An ultrasound revealed a thick walled gall bladder with multiple calculi and a normal common bile duct (CBD) and portal vein. It also detected a rounded heteroechoic lesion anterior to the portal vein with a central anechoic component, which showed flow on Doppler suggestive of an aneurysm. A contrast enhanced computed tomography scan (CECT scan) was done, which revealed similar findings suggestive of a pseudoaneurysm. A digital subtraction angiography (DSA) was then done to localize the site of the aneurysm. The selective hepatic artery angiogram showed two small pseudoaneurysms in relation to the cystic artery (Figure 1) and a normal superior mesenteric artery. As the patient had bled recently and had had an episode of acute cholecystitis (two weeks ago), embolization of the pseudoaneurysm was planned. After super selective catheterization of the cystic artery, the aneurysm was embolized using gel foam and micro coils (Figure 2). Subsequently, the patient was monitored in the intensive care unit where she remained stable haemodynamically and did not have any further episode of UGIH. A day later the patient had increasing abdominal pain and appearance of peritoneal signs localized to the RUQ of the abdomen. These clinical features suggested the possibility of gangrene of the gallbladder following embolization of the cystic artery. At laparotomy, there were dense adhesions in the gallbladder fossa. The gallbladder was inflamed, thickened, and contained multiple gallstones and blood clots. The cystic duct was obliterated with a stone that was impacted in the Hartmann's pouch. The proximal CBD was dilated (1.5 cm). There was a 3 × 3 cm pseudoaneurysm, which had ruptured into the first part of the duodenum. The small bowel and colon were filled with blood clots. A partial cholecystectomy was done as the Calot's triangle was obliterated with dense adhesions. The cut section of the gall bladder showed a grossly inflamed and oedematous wall but no evidence of gangrene. As the proximal CBD was dilated and the bilirubin had increased to 3.8 mg/dl just prior to surgery, it was explored. There were blood clots in the CBD which were removed and a T-tube was placed. The pseudoaneurysm was evacuated and the duodenal rent was closed over a 16F T-tube placed through a small duodenotomy in the lateral wall of the second part of the duodenum. A pyloric exclusion with gastrojejunostomy and feeding jejunostomy were done as the duodenal repair had been done on a severely inflammed and oedematous duodenum. Postoperatively, she was kept nil by mouth and given parenteral crystalloids, antibiotics and proton pump inhibitors. She had a leak from the duodenal closure on postoperative day 4 which was managed conservatively with maintenance of fluid and electrolyte balance, adequate drainage of the duodenal effluents, appropriate parenteral antibiotics and enteral nutrition using jejunostomy feeds. Three weeks later a gastrograffin study showed no leak from the duodenum. She was then started on oral feeds and discharged after a total hospital stay of 4 weeks. Eighteen months later on follow-up she was doing well except for an incisional hernia at the lateral edge of her operative wound.

## Discussion and Conclusions

Pseudoaneurysms arise as a consequence of visceral inflammation adjacent to the arterial wall, which leads to damage to the adventitia and thrombosis of the vasa vasorum resulting in localized weakness in the vessel wall. These are prone to rupture. Pseudoaneurysms arising from arteries of the coeliac trunk usually rupture into the bile duct or the pancreatic duct or rarely into the adjacent gastrointestinal tract.

Cystic artery related pseudoaneurysms may occur following an episode of acute cholecystitis or following cholecystectomy. However, in association with acute cholecystitis only 13 cases (Table 1) have been reported in the literature till date [2,3,5-15]. The rarity of this complication despite the high incidence of cholecystitis may be due to early thrombosis of the cystic artery in response to inflammation [5,7,11]. It is generally believed that a pseudoaneurysm develops when a large gallstone erodes the cystic artery.

Such pseudoaneurysms usually present with upper abdominal pain, jaundice and melaena. Infrequently, all three symptoms occur together and are known as Quinke's triad. Other unusual presentations include UGIH or pain with localized rupture leading to a subhepatic collection. Our patient presented with UGIH following an episode of acute cholecystitis with no jaundice. Only two patients had a similar presentation in the 13 cases reviewed [3,5].

The initial investigation for a patient suspected to have a pseudoaneurysm is ultrasonography with color Doppler. It typically demonstrates an anechoic lesion with colour flow though it is difficult to accurately localize the site of origin. The advantage of colour Doppler ultrasound in detecting pseudoaneurysms was reported by Barba *et al*. [9] and was later substantiated by Hoshino *et al*. [10] A history of cholecystitis prompted us to do an ultrasound, which raised the suspicion of a pseudoaneurysm, that was confirmed later on a CECT scan. Two of the previously reported patients in the literature without haemobilia, also had the initial diagnosis made by an ultrasound Doppler examination[6]. An UGIE usually does not reveal any lesion in the stomach and duodenum, except blood coming through the ampulla of Vater from an actively bleeding lesion. Bleeding from the papilla on side viewing endoscopy was noted in a majority (60%) of the cases reported in the literature and this helped to focus on a probable hepato-biliary source for the gastrointestinal bleeding. In our case UGIE showed a deep ulcer in the first part of the duodenum with an adherent clot. There was no obvious bleeding from the papilla at that time. These findings were interpreted, mistakenly, to suggest the lesion to be a bleeding duodenal ulcer. The diagnosis was revised following ultrasound evaluation. It is possible to miss bleeding from the papilla in patients with haemobilia as it may be intermittent in nature or simultaneous rupture into the duodenum may provide an alternative route for the blood to come out.

A CECT scan demonstrates a hyperdense lesion without contrast, which enhances in the arterial phase. It helps to confirm the diagnosis in patients with an equivocal Doppler examination before proceeding for an invasive procedure and may suggest the blood vessel involved. In our case CECT scan demonstrated typical findings suggestive of a pseudoaneurysm.

A selective hepatic artery angiography is the diagnostic modality of choice when a pseudoaneurysm is suspected. It can directly visualize the aneurysm and can be used to embolize the involved vessel. It has been suggested that in patients with cystic artery pseudoaneurysms, angiography should be done before surgery to help plan the operative procedure. Angiography helps in diagnosis, provides details of the splanchnic arterial anatomy and allows therapeutic embolization. All cases reported in the literature had had a DSA done to confirm the diagnosis before surgical intervention. Embolization was attempted in 3 patients but was successful in only one [3,14]. Angiographic embolization followed by elective cholecystectomy has also been reported [14]. Embolization may also help to stabilize the patient and convert an emergent situation to a semi-elective one. In our patient embolization was successful although the presence of peritoneal signs suggestive of gall bladder infarction led to an urgent laparotomy subsequently.

Ligation of the cystic artery pseudoaneurysm with cholecystectomy is the treatment of choice. Proximal control of the hepatic artery should be attempted first, if the patient is haemodynamically stable and there is no active bleeding. However, serious bleeding can be precipitated, when dissection is performed in an area where there is evidence of acute inflammation containing a pseudoaneurysm. In the previously reported cases surgical intervention with ligation of pseudoaneurysm was done in all except one. The presence of inflammation poses a definite risk of injury to adjacent visceral structures. In our patient the pseudoaneurysm had probably eroded into the duodenum and CBD. Hence, she required both a duodenal repair and CBD exploration. The inflamed duodenum with a large rent forced us to do a pyloric exclusion and gastrojejunostomy. These additional steps facilitated subsequent recovery despite a duodenal leak.

In conclusion, pseudoaneurysms occur rarely in patients with acute cholecystitis. Haemobilia is the most common manifestation but atypical presentations are known. Awareness of this rare complication allows specific diagnostic evaluation and timely intervention. Cholecystectomy with ligation of the pseudoaneurysm remains the treatment of choice.

## Competing interests

The author(s) declare that they have no competing interests.

## Authors' contributions

SS, SP: Operating team and drafted the manuscript, SR operating team, post-op care and helped in drafting MS: Interventional radiologist, PS and TKC: revised it critically. All authors read and approved the final manuscript.

## Pre-publication history

The pre-publication history for this paper can be accessed here:



## Figures and Tables

**Table 1:** Summary of the 10 previously reported cases and our case of cystic artery pseudoaneurysm after cholecystitis.

Sno	Age/sex	Presentation	Diagnosis		Management	Embolization
			Initial	Final		

1	64/male^2^	UGIH	USG Doppler	DSA	Ligation + cholecystectomy + CBD exploration	Not done
2	66/female^3^	UGIH	USG Doppler	DSA	Cholecystectomy + IOC	Failed
3	61/male^5^	Haemobilia	-	DSA	Cholecystectomy +CBD exploration	Not done
4	72/female^8^	Haemobilia	SVE bleeding papilla	DSA	Cholecystectomy + ligation	Not done
5	70/male^9^	Haemobilia	SVE bleeding papilla	DSA	Cholecystectomy + ligation	Failed
6	72/male^10^	Haemobilia	USG Doppler	DSA	Cholecystectomy + ligation	Not done
7	71/female^11^	Haemobilia	SVE bleeding papilla	DSA	Exploration + drainage	Not done
8	32/female^13^	Haemobilia	SVE bleeding papilla	DSA	Cholecystectomy + ligation	Not done
9	62/male^14^	Haemobilia	SVE bleeding papilla	DSA	Cholecystectomy	Done
10	43/male^15^	Haemobilia	SVE bleeding papilla	DSA	Cholecystectomy + ligation	Not done
11	43/female*	UGIH	USG Doppler	DSA	Cholecystectomy+ CBD exploration+ duodenal repair+ pyloric exclusion+ GJ	Done

• CBD: Common bile duct, DSA: Digital substraction angiography, SVE: Side viewing endoscopy, USG: Ultrasonography, UGIH: Upper gastrointestinal hemorrhage, IOC: Intra operative cholangiogram, GJ: gastrojejunostomy

• Ref 6,7, 12 not included as patient details are not available

• * Refers to the patient in the present case report
